# The social roots of student mental health: Organizational opportunity structures, social capital, and loneliness

**DOI:** 10.1016/j.ssmph.2026.101992

**Published:** 2026-10-29

**Authors:** Gunnar Voss, Philipp Pohlenz

**Affiliations:** Faculty of Humanities, Otto-von-Guericke University Magdeburg, Zschokkestr. 32, 39104, Magdeburg, Germany

**Keywords:** Social capital, Loneliness, Psychological distress, University students, Organizational opportunity structures, Student mental health, Social determinants of mental health

## Abstract

**Background:**

Individual and clinical approaches to student mental health can obscure the role of universities as social environments. Within a social-capital framework, we examine whether loneliness links peer integration and student–faculty relationship quality to later psychological distress.

**Methods:**

Three waves of the German National Educational Panel Study (NEPS SC5; *N* = 3014 enrolled students) linked relationship measures (2012) to loneliness (2013/2014) and psychological distress measured with the CDC Healthy Days instrument (2016). Indirect associations were estimated using OLS-based mediation models with 5000 bootstrap resamples, controlling for gender and age. Organizational opportunity structures are theorized but not measured.

**Results:**

Higher peer integration and student–faculty relationship quality were associated with lower loneliness. Peer integration was associated with lower distress directly (*B* = −0.40, *p* < .01) and indirectly through loneliness (*B* = −0.22, 95% CI [−0.29, −0.15]); the indirect association represented 35.3% of the total association. Student–faculty relationship quality had no significant total or direct association with distress but showed a significant indirect association through loneliness (*B* = −0.15, 95% CI [−0.22, −0.08]).

**Conclusions:**

The findings are consistent with loneliness as a pathway connecting social relationships to later psychological distress. The faculty measure is considered in light of linking social capital, while the items primarily describe perceived relationship quality. Organizational opportunities for social connection provide a theoretical extension that requires direct testing.

## Highlights

• Universities provide opportunities for social ties to develop.
• Better peer and faculty relationships were associated with less loneliness.
• Loneliness was associated with more mentally unhealthy days.
• Both relationship measures showed negative indirect associations with distress.
• Organizational pathways remain theoretical and require direct testing.

## 1 Introduction

Mental health is shaped by the social conditions in which people live. This proposition is central to public health thinking and to the World Health Organization's framework on the social determinants of health (WHO Commission on Social Determinants of Health, 2008). Among these conditions, the quality and structure of social relationships are especially consequential. Social isolation and loneliness are associated with depression, anxiety, and mortality risk (Cacioppo & Hawkley, 2003; Holt-Lunstad et al., 2015). Yet identifying a social determinant is not the same as specifying how it operates. The institutional conditions that make supportive relationships more or less likely, the resources supplied by different types of relationship, and the psychosocial pathways connecting these resources to mental health must be distinguished analytically (see Fig. 1, Fig. 2).

**Fig. 1:**
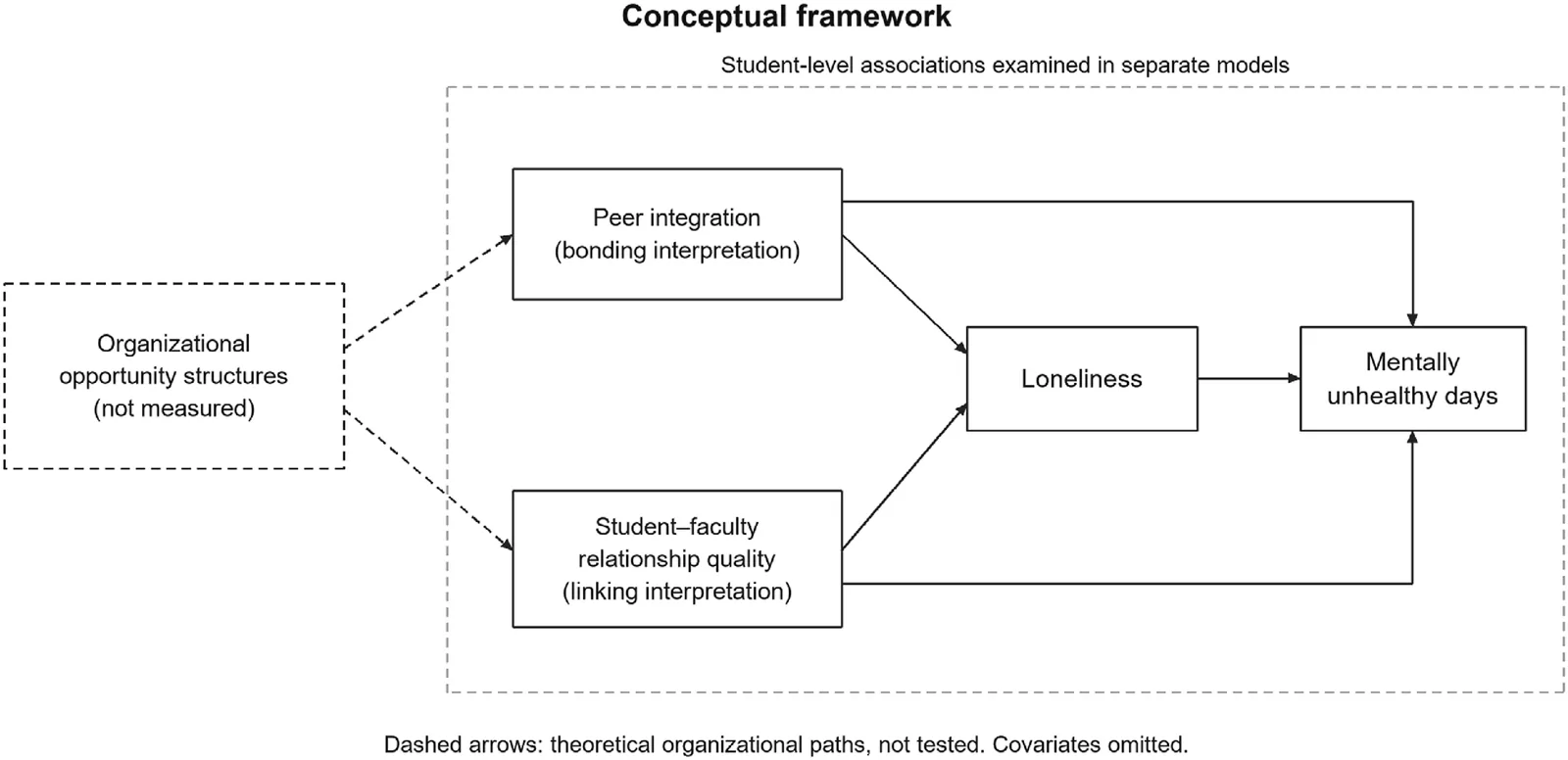
Conceptual framework. Peer integration and student–faculty relationship quality are considered in light of bonding and linking social capital, respectively; these are theoretical interpretations of integration measures. Dashed arrows identify untested organizational paths. The outlined area summarizes student-level associations examined in separate mediation models, not a jointly estimated mediation model. Covariates are omitted.

**Fig. 2:**
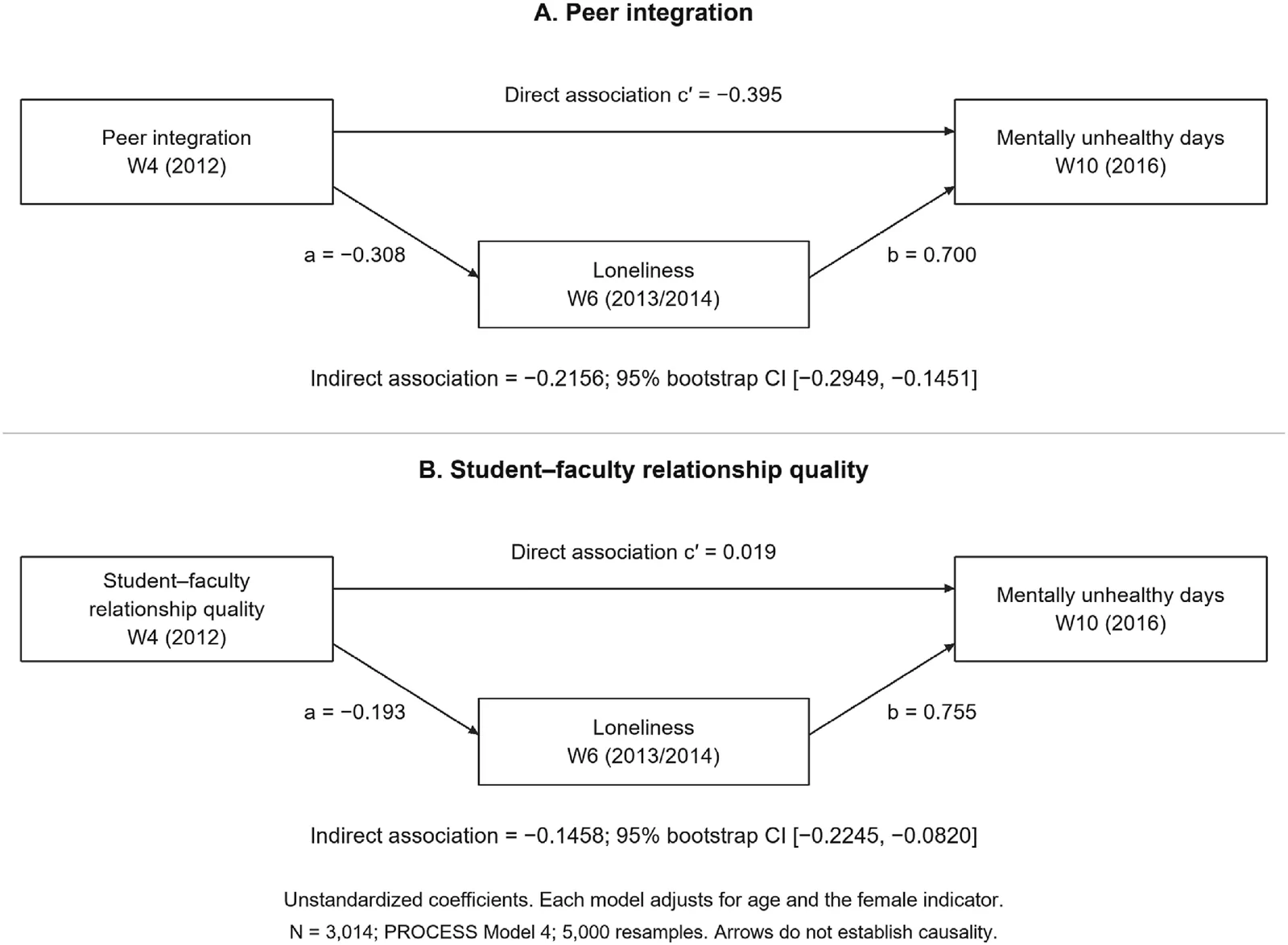
Separate linear mediation models for peer integration (A) and student–faculty relationship quality (B), N = 3014. All coefficients are unstandardized. Paths a, b and c′ correspond to Table 2; indirect associations and percentile bootstrap intervals use the verified PROCESS 4.3.1 reproduction (Model 4; 5000 resamples; seed 12345). Each model adjusts for age and the female indicator, without adjustment for the other relationship measure. Arrow style does not encode statistical significance or establish causality. These are the main linear models, not the additional nonlinear sensitivity analysis.

University students are a particularly relevant population in which to examine these connections. The expansion of higher education has made university entry a common life-course transition in many countries (OECD, 2023). Entry into higher education can involve geographic relocation, changes in established relationships, unfamiliar academic expectations, and the need to build new networks. These experiences are not simply private adjustments. They unfold within programs, courses, cohorts, teaching arrangements, timetables, campus spaces, and digital infrastructures that organize whether and how students repeatedly encounter one another.

Psychological distress is widely reported among university students, including in Australian surveys (Larcombe et al., 2016; Stallman, 2010). In a nationwide German student survey in 2017, 15.6% reported symptoms indicative of a depressive syndrome and 17.4% symptoms indicative of generalized anxiety, assessed with the PHQ-4 (Grützmacher et al., 2018). These symptom-screening estimates are not clinical diagnoses and are not directly comparable with the mentally unhealthy days measure used here. Clinical treatment and counseling remain essential for students experiencing mental health difficulties. However, a predominantly individual or service-oriented framing risks treating the university as the location in which problems appear rather than as a social setting that may shape exposure to protective and harmful conditions.

Settings-based health promotion offers a broader view. Universities are complex organizations whose everyday operations, academic practices, environments, and cultures can support or undermine health (Newton et al., 2016). The Okanagan Charter accordingly calls for health to be embedded in campus culture and everyday institutional activity rather than confined to specialist services (International Conference on Health Promoting Universities and Colleges, 2015). Complementing this institutional commitment, research on students’ perspectives identifies teaching, course design, assessment, administration, campus culture, services, and student activities as relevant to mental well-being (Baik et al., 2019). Olsson et al. (2024) reviewed approaches ranging from inclusion policies and physical environments to pedagogical practices, including peer teaching and changes to assessment. Findings varied across approaches and outcomes. A major task for research is therefore to specify credible mechanisms connecting organizational arrangements to individual mental health.

Universities also organize the occasions on which relationships can develop: repeated encounters in courses, study groups, or advising may create opportunities for connection. The empirical question is whether students' relationships with peers and teaching staff are associated with later distress through loneliness. An organizational perspective provides context for this question, although organizational arrangements are not measured here.

Using three waves of the German National Educational Panel Study (NEPS SC5), we examine these associations among 3014 students enrolled at the mediator and outcome waves. Relationship measures from 2012 precede loneliness in 2013/2014 and psychological distress in 2016. This temporal separation supports examining the proposed sequence, while the observational design leaves causal explanations unresolved.

Research has already connected university belonging with loneliness and mental health (Dao et al., 2025; Dingle et al., 2022). Our contribution is to examine peer integration and perceived faculty relationships separately in a German cohort, with relationship measures, loneliness, and mentally unhealthy days observed at successive waves. This design adds temporal separation and a comparison of relational settings, while the sensitivity analyses assess dependence on measurement and model choices. Organizational opportunity structures provide a theoretical context, rather than an additional level tested by the data.

## 2 Theoretical framework

### 2.1 Social capital: Relationships, resources, and institutional roles

Social capital directs attention to resources made available through relationships and to the norms that sustain their use (Coleman, 1988; Putnam, 2000). Bonding commonly refers to ties within groups sharing a social identity; bridging concerns connections across social groups. Szreter and Woolcock (2004) further distinguish linking social capital, which concerns relationships across institutional differences in power and authority.

This distinction matters for university students. Peers may provide companionship and mutual assistance, whereas teaching staff occupy roles involving assessment, guidance, and institutional authority. Relationships with staff therefore have a linking dimension that a bridging label alone can obscure. This is a theoretical interpretation of the relationship context, not evidence that every positive interaction with a lecturer constitutes social capital.

The empirical analysis distinguishes peer integration and student–faculty relationship quality. Peer integration is interpreted as an approximation to bonding social capital, while faculty relationship quality is considered in light of linking. Neither measure establishes which resources students actually obtain through their contacts. Relationships among students may also cross social groups, and relationships with staff may involve both support and access to opportunities. The measures therefore distinguish relational settings more securely than mutually exclusive forms of social capital.

### 2.2 Organizational opportunity structures for social capital

Relationships do not form in an institutional vacuum. Feld (1981) uses the concept of social foci to describe the social, organizational, and physical entities around which joint activities are organized. People form ties partly because they are brought together repeatedly by these foci. Small (2009) extends this reasoning through an organizational embeddedness perspective: the networks people develop and the resources they obtain depend on organizational routines, practices, participation patterns, and inducements to interact. Social capital is thus partly an outcome of institutional design.

Universities contain numerous potential foci: degree programs, introductory courses, seminars, laboratories, project groups, student residences, mentoring schemes, societies, and informal campus spaces. Their capacity to generate connection depends on how they are organized. A stable cohort with recurring small-group work creates different opportunities from an anonymous lecture sequence with changing attendance. Accessible common spaces and predictable contact points create different possibilities from fragmented timetables and largely transactional encounters. Group assessment may produce cooperation, competition, or exclusion depending on its design. Digital provision may widen access while reducing unplanned encounters. These examples illustrate organizational opportunities; they do not establish that any single arrangement will necessarily produce beneficial ties.

An opportunity-structure perspective avoids assigning full responsibility for integration to individual students. Students differ in time, financial resources, caring responsibilities, health, language, residence, and familiarity with academic culture. The same invitation to “get involved” is therefore not equally useable by everyone. Institutional arrangements can lower or raise the cost of participation and can distribute opportunities for repeated interaction unevenly. This makes social capital relevant to both health and inequality.

This perspective complements whole-university approaches to health promotion, which connect health to interacting organizational domains (Dooris et al., 2020; Newton et al., 2016). It specifies repeated encounters and access to relational resources as processes through which those domains might matter.

### 2.3 Social capital, social integration, and mental health

Social-epidemiological accounts connect relationships to health through support, participation, social influence, and access to resources (Berkman & Glass, 2000; Kawachi & Berkman, 2001). These processes need not coincide. Having contacts does not establish that support is available, and receiving academic advice need not resolve loneliness.

Educational research similarly distinguishes dimensions of integration (Tinto, 1987) and examines the resources accessible through educational relationships (Dika & Singh, 2002). Stanton-Salazar's (1997) account of institutional agents, developed in research on minority children and youths, provides a related conceptual perspective on access to resources through people occupying institutional roles. Its application to university teaching relationships requires attention to what the available measures capture.

A systematic review and meta-analysis found a positive association between university belonging and well-being, alongside substantial heterogeneity and predominantly high risk of bias (van Kessel et al., 2025). This supports the relevance of belonging without establishing causal protection. A scoping review of 21 studies in health professions education likewise linked supportive faculty interactions with better mental-health outcomes, while identifying gaps in measurement and theory (Bulan et al., 2025). Its disciplinary scope limits generalization to all university students. In a survey of graduate students in South Korea, Kim et al. (2023) found that faculty interactions were associated with greater belonging and lower academic stress, including an indirect association through belonging. This cross-sectional study concerns a different population and outcome, but supports considering inclusion as one possible link between faculty relationships and distress. Dao et al. (2025) examined belonging and mental health across the first college year in a small US sample; their baseline findings linked social and academic belonging with lower depression and greater well-being. Belonging and institutional support should therefore be distinguished from the relationship measures themselves.

Our peer items concern building contacts, discussing academic questions, and having many contacts with students. The faculty items concern recognition, getting along with staff, fair treatment, and staff interest. They describe perceived relationships rather than network structure, reciprocity, or resources mobilized. We consequently use “student–faculty relationship quality” as the empirical label. Interpreting this measure through linking social capital does not make it a comprehensive measure of that construct.

### 2.4 Loneliness as a proximate pathway

Loneliness is the subjective discrepancy between desired and experienced social connectedness (Perlman & Peplau, 1981). It is related to mental-health outcomes across populations. Proposed mechanisms include heightened sensitivity to social threat, impaired self-regulation, and physiological stress responses (Hawkley & Cacioppo, 2010). Loneliness is not synonymous with having few contacts: a person can be surrounded by others and still experience their relationships as insufficient.

Student evidence also distinguishes connectedness from loneliness. Across three first-year cohorts at an Australian university, Dingle et al. (2022) found that loneliness was associated with greater psychological distress, while university belonging was associated with better mental health. The comparison involved different cohorts before and during the pandemic, rather than repeated observations of the same students. It supports the relevance of these constructs while illustrating why context and design matter when interpreting their associations.

Peer contacts may provide companionship and everyday support, making loneliness a plausible intervening experience. For faculty relationships, our proposed connection is more specific: recognition, fair treatment, and interest from staff may communicate that students are accepted within the university. Such experiences could matter for loneliness even when lecturers are not sources of friendship. This interpretation concerns perceived inclusion across institutional roles; it does not assume that access to academic information alone reduces loneliness. The items cannot distinguish this proposed process from perceived support or more general evaluations of university life.

The study aims to examine the longitudinal associations of peer integration and student–faculty relationship quality with subsequent loneliness and mentally unhealthy days, including indirect associations through loneliness. The mediation models represent this theoretically ordered sequence statistically; temporal separation does not eliminate confounding, selection, or reciprocal influence. The following hypotheses guide the analysis:

H1 Higher peer integration is associated with fewer later mentally unhealthy days (total association).

H2 Higher peer integration is associated with fewer later mentally unhealthy days indirectly through lower loneliness.

H3 Higher student–faculty relationship quality is associated with lower later distress indirectly through lower loneliness. The direct and total associations are examined alongside this indirect association; a non-significant estimate does not establish the absence of an association or a fully mediated process.

## 3 Methods

### 3.1 Data

This study is a secondary analysis of data from Starting Cohort 5 (SC5) of the German National Educational Panel Study (NEPS), using the NEPScomp simplified panel version (v1.0.0; https://doi.org/10.5157/NEPScomp:SUF:1.0.0). SC5 began with students entering higher education in Germany in 2010 and followed participants across subsequent educational and employment trajectories. The full panel contains 17,909 participants and 19 survey waves through 2022.

The original SC5 sampling design covered public universities, public universities of applied sciences, and private higher education institutions, with oversampling of teacher-education students and students at private institutions (FDZ-LIfBi, 2020). This describes cohort recruitment, not the proportions of institution types in the present analytic sample. Our analyses were unweighted and do not provide nationally representative prevalence estimates.

Among 17,909 participants represented in the NEPScomp SC5 file, 3550 were documented as enrolled at both wave 6 and wave 10. Of these, 536 (15.1%) lacked at least one core analysis variable, leaving 3014 participants. Complete information refers here to the two relationship scores, loneliness, mentally unhealthy days, and the demographic covariates; individual scale items could be missing as described below. Enrollment at the two focal waves does not establish uninterrupted enrollment between them. The sample size followed from these eligibility and data-availability criteria, rather than an a priori power calculation.

Relationship measures were collected at wave 4 (2012), loneliness at wave 6 (2013/2014), and mentally unhealthy days at wave 10 (2016), following the NEPS study overview (Leibniz Institute for Educational Trajectories, 2017). The intervals reflect the panel survey schedule and availability of the selected measures, rather than an established timescale for the proposed pathway. These observations precede the COVID-19 pandemic and allow the associations to be examined without pandemic-related disruption of university life. Their transferability to contemporary higher education nevertheless requires examination.

### 3.2 Measures

**Psychological distress (outcome).** The NEPS Healthy Days item (t521051) asks about the number of days in the previous 30 days on which respondents felt unwell because of their mental state, explicitly including stress, depression, and mood. Responses range from 0 to 30, with higher values indicating greater distress. This item belongs to the CDC Health-Related Quality of Life “Healthy Days” approach (Moriarty et al., 2003). It measures perceived mentally unhealthy days, not a diagnosis of depression or anxiety.

**Peer integration.** Three NEPS social-integration items provide an approximation to bonding social capital: “I have successfully built contacts with other students,” “I discuss field-specific questions with fellow students,” and “I have many contacts with students.” Items use a four-point scale from 1 (does not apply at all) to 4 (fully applies). A mean score was calculated; Cronbach's α = .84 at wave 4.

**Student–faculty relationship quality.** Four items capture perceived relationships with teaching staff: “I receive recognition from the teaching staff,” “I get along well with the teaching staff,” “I am treated fairly by the teaching staff,” and “The teaching staff shows interest in me.” Items use the same four-point response format. A mean score was calculated; Cronbach's α = .79 at wave 4.

For both relationship measures, the score was the mean of the available items, requiring at least one valid response. Each scale had 2984 participants with complete items; 2954 had all seven items. The reported reliability estimates use the complete items for each scale. We examined sensitivity to partial item completion by repeating the models among the 2954 participants with all seven responses.

**Loneliness (mediator).** At wave 6, participants rated the statement “I often feel lonely” (German original: “Ich fühle mich oft einsam”; t527017). Responses ranged from 1 (does not apply at all) to 5 (applies completely), with 3 indicating partial agreement. Higher scores indicate greater agreement with this statement. This is an agreement scale, not a scale ranging from “never” to “constantly”; category groupings are not interpreted as prevalence thresholds.

**Covariates.** Models include the female indicator and age. Age was calculated as 2010 minus year of birth, using the demographic information from wave 1; it therefore describes age in the panel-entry year rather than exact age on the interview date.

### 3.3 Analytical strategy

The analysis examines whether loneliness statistically accounts for part of the longitudinal association between social capital and psychological distress. Predictors were measured at wave 4 (2012), the mediator at wave 6 (2013/2014), and the outcome at wave 10 (2016). This sequence establishes temporal ordering in measurement, but does not on its own identify a causal effect.

Indirect associations were estimated with OLS-based mediation models using PROCESS Model 4 for SPSS (Hayes, 2022, 2025). For each relationship measure, the model estimates the total association with distress (*c*), the association with loneliness (*a*), the association between loneliness and distress conditional on the relationship measure (*b*), and the direct association between the relationship measure and distress conditional on loneliness (*c′*). The indirect association is the product *a* × *b*. Its uncertainty was assessed using percentile bootstrap 95% confidence intervals based on 5000 resamples. Because the sampling distribution of a product term is generally non-normal, the bootstrap interval is the primary inferential criterion for the indirect association (Preacher & Hayes, 2008). Path-specific regressions are reported to aid interpretation. Following contemporary mediation analysis, an indirect association can be estimated and interpreted even when the total association is not statistically significant (Hayes, 2022).

The two mediation models were fitted separately, each adjusting for age and the female indicator; their indirect estimates are not mutually adjusted for the other relationship measure. A joint outcome model included both relationship measures and loneliness with the same covariates. Supplementary Fig. S1 sets out the assumed temporal and confounding structure. These linear analyses were conducted in SPSS version 31 with PROCESS version 4.3.1.

We examined whether the findings depended on prior general health or partial completion of the relationship items. General health at wave 1 was included as two indicators (good; fair to very poor), with very good health as the reference, in both equations of each mediation model. This retained all 3014 participants. In the NEPS scientific-use data available to us, we did not identify a measure of mentally unhealthy days collected before the relationship measures. General health therefore provides an additional adjustment, not a substitute for prior psychological distress. A separate analysis required all seven relationship items (N = 2954).

We also checked the component associations using models that allow for the five categories of loneliness and the skewed count of mentally unhealthy days. Multinomial models were used for loneliness after initial ordinal models showed departures from the proportional-odds assumption. Negative-binomial outcome models included loneliness as four category indicators. We then combined the multinomial and negative-binomial models to estimate indirect differences in expected unhealthy days for relationship scores of 2.5 versus 3.5, averaging predictions over the observed covariates. We report both contrasts obtained by holding the relationship score in the outcome model at 2.5 or 3.5. Uncertainty was assessed with 5000 paired person-level bootstrap resamples, refitting both component models in each resample (seed 20260913). The combined calculation was implemented in Python; its point estimates were also reproduced in SPSS. Supplementary Table S4 describes the calculation and reports all contrasts. The negative-binomial model also does not explicitly impose the outcome's upper bound of 30 days.

OpenAI Codex (OpenAI) assisted with analysis-code development and debugging. The statistical calculations were executed by the documented SPSS and Python routines. Reproducible code and numerical checks are documented in the supplement; AI-generated code was not treated as independent validation.

## 4 Results

### 4.1 Descriptive statistics

Table 1a presents descriptive statistics for the analytical sample (Mean, Standard Deviation, Min-Max) while Table 1b provides the zero-order correlations among the core variables in the study.

**Table 1a:** Descriptive statistics (N = 3014 students enrolled at waves 6 and 10).

Variable	*M*	*SD*	Min	Max
Peer integration (wave 4, 1–4)	3.09	0.68	1	4
Student–faculty relationship quality (wave 4, 1–4)	3.09	0.49	1	4
Loneliness (wave 6, 1–5)	2.33	1.02	1	5
Mentally unhealthy days (wave 10, 0–30)	3.34	4.97	0	30
Female (0/1)	0.57	—	0	1
Age in 2010	20.83	3.38	16	64

**Table 1b:** Zero-order correlations among the core study variables.

Variable	1	2	3	4
1. Peer integration	—			
2. Student–faculty relationship quality	0.186	—		
3. Loneliness	−0.202	−0.092	—	
4. Mentally unhealthy days	−0.081	−0.010	0.155	—

The distribution of psychological distress was right-skewed, as is common for Healthy Days data in non-clinical populations. Of the sample, 37.3% reported no mentally unhealthy day in the previous month and 47.0% reported between one and six days. A further 9.4% reported 7–13 mentally unhealthy days and 6.4% reported at least 14 days, a threshold frequently used to indicate frequent mental distress (Moriarty et al., 2003). The mean was 3.34 days (*SD* = 4.97).

Responses to “I often feel lonely” were distributed as follows: 596 (19.8%) selected “does not apply at all,” 1354 (44.9%) “tends not to apply,” 625 (20.7%) “partly applies,” 343 (11.4%) “tends to apply,” and 96 (3.2%) “applies completely.” These are agreement categories rather than frequencies of loneliness.

Among participants enrolled at both focal waves, excluded participants had a similar observed mean age (20.83 years) but slightly higher mean loneliness scores (2.38 versus 2.33) and mentally unhealthy days (3.73 versus 3.34). The proportion classified as female was 52.6% among excluded participants and 57.1% among included participants. Peer and faculty scores were observed for only 57 and 67 excluded participants, respectively. Supplementary Table S1 reports valid sample sizes and missingness patterns; these comparisons cannot rule out selection bias.

Pearson correlations; N = 3014 for all pairs. All two-sided p < .001 except the faculty–unhealthy-days correlation (p = .577). These are unadjusted associations. Spearman rank correlations are reported in Supplementary Table S3.

### 4.2 Indirect associations through loneliness

We estimated separate mediation models for peer integration and student–faculty relationship quality, adjusting each model for age and gender. Table 2 (Panel A & B) summarizes the direct, indirect, and total associations with later mentally unhealthy days (see Table 3).

**Table 2:** Relationship measures (wave 4), loneliness (wave 6), and mentally unhealthy days (wave 10).

Panel A: Peer integration				
Path	*B*	*SE*	β	*p*
*c*: Peer integration → Mentally unhealthy days (total)	−0.611	0.132	−0.084	<0.001
*a*: Peer integration → Loneliness	−0.308	0.027	−0.206	<0.001
*b*: Loneliness → Mentally unhealthy days (controlling peer integration)	0.700	0.089	0.143	<0.001
*c′*: Peer integration → Mentally unhealthy days (controlling Loneliness)	−0.395	0.133	−0.054	<0.01
Indirect association (*a* × *b*)	−0.216	0.038		
95% bootstrap CI (5000 resamples)	[−0.295, −0.145]			
Indirect/total association	35.3%			

Panel B: Student–faculty relationship quality				
Path	*B*	*SE*	β	*p*
*c*: Faculty relationships → Mentally unhealthy days (total)	−0.126	0.184	−0.012	n.s.
*a*: Faculty relationships → Loneliness	−0.193	0.038	−0.092	<0.001
*b*: Loneliness → Mentally unhealthy days (controlling faculty relationships)	0.755	0.087	0.155	<0.001
*c′*: Faculty relationships → Mentally unhealthy days (controlling Loneliness)	0.019	0.183	0.002	n.s.
Indirect association (*a* × *b*)	−0.146	0.037		
95% bootstrap CI (5000 resamples)	[−0.225, −0.082]			

*Note.* The indirect-association SE is the bootstrap standard error (5000 resamples; seed 12345). *N* = 3014; *R*^2^ = 0.047; gender and age controlled throughout. Mediator models (loneliness regressed on the relationship measure and covariates) produced *R*^2^ = 0.042 for peer integration and 0.009 for faculty relationships. Total-association models (distress regressed on the relationship measure and covariates) produced *R*^2^ = 0.030 and 0.024, respectively.

*Note. N* = 3014; *R*^2^ = 0.050; gender and age controlled throughout. *B* = unstandardized coefficient; β = standardized coefficient. For indirect associations in Table 2, the SE column reports the bootstrap standard error, based on 5000 resamples (seed 12345).

**Table 3:** Full outcome model including both relationship measures and loneliness.

Predictor	*B*	*SE*	β	*p*
Peer integration (wave 4)	−0.410	0.135	−0.056	<0.010
Student–faculty relationship quality (wave 4)	0.115	0.185	0.011	n.s.
Loneliness (wave 6)	0.703	0.089	0.144	<0.001
Female	1.505	0.179	0.150	<0.001
Age	0.054	0.026	0.037	<0.050

*Note. N* = 3014; *R*^2^ = 0.050. *B* = unstandardized coefficient; β = standardized coefficient.

Peer integration showed a negative total association with later distress (*B* = −0.611, *p* < .001; β = −0.084), supporting H1. It was negatively associated with loneliness at the subsequent measurement wave (path *a*: *B* = −0.308, *p* < .001), and loneliness was positively associated with distress conditional on peer integration (path *b*: *B* = 0.700, *p* < .001). The indirect association was *B* = −0.216 (95% CI [−0.295, −0.145]), corresponding to 35.3% of the total association. The direct association remained statistically significant (*B* = −0.395, *p* < .01). These findings support H2 and are consistent with an indirect association through loneliness alongside a remaining direct association.

Student–faculty relationship quality did not show a statistically significant total association with distress (*B* = −0.126, 95% CI [−0.488, 0.235]). It was nevertheless negatively associated with loneliness, with a descriptively smaller standardized coefficient than peer integration (path *a*: β = −0.092 compared with β = −0.206). Conditional on student–faculty relationship quality, loneliness was positively associated with distress (path *b*: *B* = 0.755, *p* < .001). The direct association between student–faculty relationship quality and distress was close to zero (*B* = 0.019, 95% CI [−0.339, 0.378]), while the bootstrapped indirect association excluded zero (*B* = −0.146, 95% CI [−0.225, −0.082]). The negative indirect association is consistent with H3. The difference between the peer and faculty estimates has not been formally tested. It is more precisely described as a significant indirect association in the absence of significant total and direct associations than as proof of “full mediation.” The direct and total confidence intervals remain compatible with both negative and positive associations; they do not establish practical equivalence to zero.

### 4.3 Full outcome model

In the joint outcome model, peer integration remained negatively associated with mentally unhealthy days (*B* = −0.410, *p* < .01); the faculty coefficient was not statistically significant (*B* = 0.115). Loneliness (β = 0.144) and the female indicator (β = 0.150) had the largest standardized coefficients. This model adjusts for both relationship measures, unlike the separate mediation models, and its coefficients should not be combined with their indirect estimates to calculate total associations. The largest variance inflation factor was 1.092, providing no indication of problematic multicollinearity.

### 4.4 Sensitivity analyses

After adjustment for wave-1 general health, the indirect estimates were smaller but remained negative: −0.174 days for peer integration (95% bootstrap CI [−0.244, −0.112]) and −0.110 for faculty relationship quality [−0.178, −0.056]. Requiring all seven relationship items also preserved the pattern (peer integration: −0.213 [−0.291, −0.143]; faculty relationships: −0.140 [−0.218, −0.076]). Supplementary Table S2 reports these estimates alongside the main models. These findings do not establish equivalence between specifications or remove unmeasured confounding.

The component checks were also consistent in direction. Higher relationship scores were associated with lower odds of each higher loneliness category relative to the lowest category in the multinomial models. In the negative-binomial outcome models, higher loneliness categories were associated with higher expected counts of unhealthy days; the peer coefficient was negative, while the faculty coefficient remained uncertain. The uncertainty in the latter should not be interpreted as proof of an absent association.

The combined nonlinear models yielded negative indirect differences for both relationship measures. For peer integration, the estimates were −0.236 days (95% CI [−0.319, −0.162]) and −0.216 [−0.294, −0.148], holding the outcome-model score at 2.5 and 3.5, respectively. Corresponding faculty estimates were −0.129 [−0.217, −0.055] and −0.132 [−0.225, −0.055]. Thus, the direction of the indirect associations persisted under the alternative models. These contrasts use different functional assumptions from the linear product estimates and do not establish causal mediation.

## 5 Discussion

### 5.1 Main findings

This study examined whether loneliness statistically links peer and faculty relationships to later psychological distress. Two findings stand out. First, peer integration was associated with both lower loneliness and lower later distress. Its indirect association through loneliness amounted to 35.3% of the estimated total association, while a negative direct association remained. This proportion describes the fitted statistical decomposition, not the share of distress causally explained by loneliness. Second, the pattern for student–faculty relationship quality differed descriptively. Its total and direct associations with distress were not statistically significant, although the bootstrapped indirect association through loneliness excluded zero.

The patterns may reflect different relational functions, but the mechanisms were not separately measured and differences between coefficients were not formally tested. The uncertain direct and total faculty associations should not be interpreted as evidence that these relationships are unimportant.

The sequence of measurements is consistent with the proposed pathway, but does not demonstrate changes caused by relationships or loneliness. It provides a basis for more direct investigation through repeated measurements and intervention research.

### 5.2 Differentiating relationships and their possible functions

The faculty findings invite a more precise interpretation than the original bridging label allowed. Students and lecturers differ in institutional authority, making linking a relevant conceptual perspective (Szreter & Woolcock, 2004). The observed indirect association may reflect recognition and inclusion across these roles. It does not demonstrate that students gained institutional resources or that such access reduced loneliness. Perceived support remains an overlapping explanation that our items cannot separate.

For peers, the association is compatible with companionship and mutual assistance, but the analysis does not directly identify these mechanisms. Nor does it imply that denser networks are invariably beneficial: bonding may support insiders while excluding others or imposing demands (Portes, 1998). Practical implications therefore concern accessible, supportive relationships rather than maximizing contact counts.

### 5.3 From individual networks to organizational opportunity structures

The findings raise an organizational question: which arrangements make supportive relationships more likely, and for whom? Feld's (1981) focus theory and Small's (2009) organizational embeddedness perspective suggest examining how programs organize repeated encounters and shared activities. Stable cohorts, recurring small groups, and accessible common spaces are plausible settings for such contact. The present analysis does not test these arrangements; it identifies student-level associations that future studies can connect to institutional conditions.

### 5.4 Designing opportunities rather than prescribing sociability

Universities can provide occasions for connection, but cannot prescribe friendship. Practical proposals therefore need evidence about both participation and outcomes.

A broader review of reviews mapped interventions across learning, living environments, and support, finding uneven coverage and mostly limited or mixed evidence (Upsher et al., 2025). This strengthens the case for evaluating educational conditions alongside other university activities, while cautioning against assuming that a whole-university strategy is already established as an effective intervention. The review focused on complementary approaches and excluded psychological interventions; it therefore does not compare these approaches with clinical treatment.

Possible actions should be matched to the function sought. Arrangements intended to support peer connection include cohort structures, facilitated study groups, peer mentoring, collaborative learning, and recurring low-threshold activities. Arrangements intended to support recognition and access to institutional resources include structured advising, reliable feedback, accessible office hours, and staff contact embedded in the curriculum. Some practices may support both. Their effectiveness will depend on implementation and on whether students with fewer resources can actually participate.

A more direct example is The Connection Project, a facilitated group program evaluated in a randomized trial with 438 first-year and transfer students at one US university (Costello et al., 2022). Compared with a waitlist, participants reported greater belonging and lower loneliness and depressive symptoms immediately after the program. The pandemic setting, short follow-up, and absence of an active comparison group limit conclusions about transferability and which program elements produced the benefits. The study supports evaluating organized peer contact without establishing our proposed mediation pathway.

The reviewed interventions provide examples to evaluate rather than a universal package. Olsson et al. (2024) describe pass/fail grading with formative feedback and teaching that treats mistakes as opportunities for learning. Such practices may alter competition, trust, and relationships, but these mechanisms were not tested in our analysis. Evidence was mixed: the review also reports a randomized peer-teaching study without a reduction in student anxiety. Future evaluations should measure both the intended relational changes and subsequent health outcomes.

### 5.5 Population health and equity implications

The association between loneliness and later distress supports examining social connection alongside clinical need. Counseling and clinical care remain crucial, while a population-health perspective also asks how the conditions of everyday study distribute opportunities for connection. The single agreement item used here does not establish a prevalence of frequent loneliness.

Opportunity structures also have an equity dimension. Students' ability to use optional social activities varies with paid work, commuting, disability, caring obligations, financial constraints, language, and prior familiarity with university culture. If opportunities for connection are confined to residential life, evening activities, or voluntary extracurricular engagement, they may systematically miss students who could benefit most. Embedding recurring contact in ordinary curricula may distribute opportunities more broadly, although curricular arrangements can also reproduce exclusion if poorly designed.

Female students reported more mentally unhealthy days than male students, and gender had one of the largest standardized coefficients in the full outcome model. The analysis does not establish why this difference occurs or whether the social-capital pathways vary by gender. Future research should test moderated pathways and examine additional dimensions of inequality rather than treating the average association as universal.

### 5.6 Limitations and future research

Several limitations define the scope of the findings. First, the integration items approximate relational aspects of social capital without measuring network structure, reciprocity, or the resources obtained. The faculty measure also overlaps with perceived institutional support. Future instruments should distinguish relationship quality, differences in authority, and access to resources, rather than assign bonding, bridging, or linking labels solely from the identity of the people involved.

Second, loneliness was measured with a single item at one wave. Brief measures are useful in large surveys (Hughes et al., 2004), but the single item used here does not distinguish social from emotional loneliness. Measurement at only one wave also prevents analysis of loneliness trajectories. Measurement error may affect the estimated associations.

Third, inclusion required enrollment at waves 6 and 10 and complete core analysis variables. The comparison with the 536 excluded participants within this enrollment group was limited by extensive missingness in their relationship scores. It does not address selection associated with leaving higher education or having unknown enrollment status. Neither these comparisons nor the direction of observed differences establish that the estimates are unbiased or conservative.

Fourth, unmeasured confounding and reciprocal relationships remain possible. Prior mental health may influence later social integration and loneliness. Although the variables are measured in temporal sequence, temporal ordering does not remove these threats to causal inference. Parental education, financial resources, paid work alongside study, and institutional sector were not included in the models. NEPS contains relevant socioeconomic information, but the present analysis does not establish the sample's composition or the consistency of associations across these characteristics. Financial and time constraints may shape both opportunities for relationships and distress, leaving residual confounding and social heterogeneity unresolved. Models with repeated measures of each construct, baseline outcome adjustment, fixed effects, cross-lagged comparisons, or stronger identification strategies would improve the evidence.

Fifth, psychological distress is measured with a single Healthy Days item. The measure captures perceived mental unhealth over the previous month but is not a clinical diagnosis and does not distinguish depression, anxiety, or other conditions. Findings should not be generalized to specific disorders.

Sixth, the proposed organizational level is not measured. We cannot determine which program, faculty, or university arrangements produced the observed differences in social capital. The institutional propositions in this paper are therefore a research agenda grounded in organizational and settings-based theory, not an empirical result of the NEPS analysis. Multilevel data should connect student measures to program-level features such as cohort stability, group size, contact intensity, pedagogical format, timetable structure, campus participation, and accessibility. Such studies should also examine whether opportunity structures operate differently across social groups.

Finally, the data were collected between 2012 and 2016, allowing these relationships to be examined outside the specific disruptions of the COVID-19 pandemic. This historical context does not remove other sources of stress or establish that the associations generalize to current students. Replication with recent data should examine whether the findings persist under contemporary study conditions.

## 6 Conclusion

Peer integration and perceived relationships with teaching staff were associated with later loneliness, which in turn was associated with mentally unhealthy days. The peer measure showed negative direct and indirect associations with distress; the faculty measure showed a negative indirect association, with uncertain direct and total associations. These observational findings identify relationships worth examining in research on student mental health, without establishing causal effects. Their relevance to current university settings requires replication beyond this pre-pandemic cohort.

They also illustrate why conceptual precision matters for interpreting secondary data. Faculty relationships span institutional roles and may be better understood through linking than through bridging alone. The unresolved issue is how this established distinction can be represented in measures that also capture support and inclusion. This is consequential for interpretation: similar item responses may indicate recognition across institutional roles, access to resources, or a broader sense of support. Future research should connect relationship quality to resources actually available and used, and test how university arrangements shape these processes.

## CRediT authorship contribution statement

**Gunnar Voss:** Writing – original draft, Formal analysis, Conceptualization. **Philipp Pohlenz:** Writing – review & editing, Supervision, Methodology, Data curation, Conceptualization.

## Ethics declaration

Written informed consent to take part in the study and to publish the article has been obtained from all participants or their legal representatives. The privacy rights of participants have been observed.

This study was performed in compliance with relevant laws, regulatory frameworks and guidelines where the research took place. Ethics committee approval was not required under relevant laws and institutional guidelines. The data were provided as a scientific use file by the Institute for Education Trajectories. They are part of a large-scale assessment study, the National German Education Panel Study. The data collection under this study is carried out in line with all ethical and data protection related obligations.

## Ethics statement

This study used anonymized secondary data from the NEPS SC5 panel, collected under ethical approval by the Leibniz Institute of Education Trajectories [Leibniz Institut für Bildungsverläufe] LIfBi. No additional ethical approval was required for this secondary analysis.

## Declaration of generative AI use

During the preparation and revision of this work, the authors used generative AI tools (Anthropic Claude and OpenAI Codex) for language editing, argument structuring, and manuscript formatting. OpenAI Codex also assisted with analysis-code development and debugging. AI-generated suggestions were not treated as independent validation of the analyses. The authors take responsibility for the analytical decisions, reported results, source selection, and interpretation.

## Funding

This work was supported by the German Federal Ministry of Research, Technology and Space (BMFTR) as part of the HoPsy project (Hochschule und Psychische Gesundheit) [grant number 16RBM2006A-B]. The funder had no role in the study design, analysis or interpretation of data, writing of the report, or decision to submit the article for publication.

## Declaration of competing interest

The authors declare the following financial interests/personal relationships which may be considered as potential competing interests: Philipp Pohlenz reports financial support was provided by Federal Ministry of Research Technology and Space. If there are other authors, they declare that they have no known competing financial interests or personal relationships that could have appeared to influence the work reported in this paper.

## Data Availability

The NEPS SC5 data used in this study are available to eligible researchers from the Leibniz Institute for Educational Trajectories (LIfBi), subject to a Scientific Use File agreement (NEPScomp v1.0.0; https://doi.org/10.5157/NEPScomp:SUF:1.0.0). The authors are not permitted to redistribute the data. Analysis materials are available from the corresponding author upon reasonable request, subject to the applicable data-use conditions.
