# Supplementary material for: The social roots of student mental health: Organizational opportunity structures, social capital, and loneliness
Source: SSM Popul Health. 2026 Oct 29;36:101992. doi: 10.1016/j.ssmph.2026.101992 (PMC13744898; doi:10.1016/j.ssmph.2026.101992)
Supplement: Supplementary file 1 [file mmc1.docx]

Supplementary material

# Table S1. Selection within participants documented as enrolled at waves 6 and 10

There were 3,550 participants in this group: 3,014 included and 536 excluded because of missing core analysis variables. Values below are observed-case summaries within each group.

| **Variable** | **Included valid n** | **Included mean (SD)** | **Excluded valid n** | **Excluded mean (SD)** |
| --- | --- | --- | --- | --- |
| Peer integration | 3,014 | 3.0865 (0.68121) | 57 | 3.0526 (0.75538) |
| Student–faculty relationship quality | 3,014 | 3.0932 (0.48608) | 67 | 2.9950 (0.49341) |
| Loneliness agreement score | 3,014 | 2.3328 (1.01813) | 457 | 2.3764 (1.03583) |
| Mentally unhealthy days | 3,014 | 3.3368 (4.96844) | 535 | 3.7308 (5.45997) |
| Age in 2010 | 3,014 | 20.8311 (3.37525) | 536 | 20.8321 (3.06835) |

The female indicator was 1 for 1,720/3,014 (57.1%) included participants and 282/536 (52.6%) excluded participants. Loneliness is an ordinal agreement item; the mean is provided only as a descriptive summary. It was measured at wave 6, not before the relationship measures.

| **Missing core variables among excluded participants** | **n** |
| --- | --- |
| Peer integration only | 10 |
| Both relationship scores only | 446 |
| Loneliness only | 56 |
| Both relationship scores and loneliness | 23 |
| Mentally unhealthy days only | 1 |
| Total | 536 |

Thus, peer integration was missing for 479 (89.4%) and faculty relationship quality for 469 (87.5%) excluded participants. The corresponding observed-score comparisons are highly restricted. Missingness patterns do not identify whether missing values arose from wave nonparticipation, questionnaire routing, or item nonresponse. These comparisons cannot rule out selection bias and do not address selection associated with the enrolment requirement.

# Table S2. Indirect associations in the linear models

| **Specification** | **N** | **Peer estimate** | **BootSE** | **95% percentile CI** | **Faculty estimate** | **BootSE** | **95% percentile CI** |
| --- | --- | --- | --- | --- | --- | --- | --- |
| Main model | 3,014 | −0.2156 | 0.0377 | [−0.2949, −0.1451] | −0.1458 | 0.0367 | [−0.2245, −0.0820] |
| Additional general-health adjustment | 3,014 | −0.1736 | 0.0335 | [−0.2439, −0.1115] | −0.1100 | 0.0316 | [−0.1782, −0.0560] |
| All seven relationship items complete | 2,954 | −0.2133 | 0.0378 | [−0.2908, −0.1434] | −0.1400 | 0.0361 | [−0.2179, −0.0763] |

Separate PROCESS 4.3.1 Model 4 analyses, adjusted for age and the female indicator, with 5,000 bootstrap resamples and seed 12345. Estimates are unstandardized indirect associations in mentally unhealthy days per one-point relationship-score difference. General-health categories were very good (reference; n = 1,544), good (n = 1,222), and fair to very poor (n = 248). The latter two indicators entered both equations. The complete-item analysis changes the sample as well as the completeness requirement; it does not isolate an effect of scoring rules.

# Table S3. Rank correlations among the core study variables

| **Variable** | **1** | **2** | **3** | **4** |
| --- | --- | --- | --- | --- |
| 1. Peer integration | — |  |  |  |
| 2. Student–faculty relationship quality | .176 | — |  |  |
| 3. Loneliness | −.193 | −.093 | — |  |
| 4. Mentally unhealthy days | −.091 | −.027 | .178 | — |

Spearman correlations; N = 3,014 for all pairs. All two-sided p < .001 except the faculty–unhealthy-days correlation (p = .139). These unadjusted rank correlations complement the Pearson correlations in Table 1b; they are not estimates of indirect associations.

# Table S4. Combined nonlinear sensitivity analysis

Each relationship measure was analysed separately in the same 3,014 participants. A multinomial logistic model estimated the probabilities of the five loneliness categories (reference: category 1). An NB2 outcome model with log link included four loneliness-category indicators. Both models adjusted for the female indicator and baseline age. No exposure-by-loneliness interaction was included. Earlier general health was not included in this specification; that adjustment is reported separately in Table S2. The negative-binomial distribution does not impose the upper limit of 30 days.

Let F(a,b) denote the sample average of the sum, over loneliness categories m, of the predicted mean days mu(a,m,C) multiplied by the predicted category probability p(m|b,C). C denotes the observed covariates. We compared x0=2.5 with x1=3.5, both within the observed 1–4 relationship-score range. This is a model-based one-point contrast, not an observed within-person change. The two indirect contrasts hold the outcome-model score at x0 or x1; differences between them can arise from the log link without an explicit interaction term.

| **Relationship measure** | **Contrast** | **Estimate (days)** | **Bootstrap SE** | **95% percentile CI** |
| --- | --- | --- | --- | --- |
| Peer integration | Indirect at x0: F(x0,x1) − F(x0,x0) | -0.2365 | 0.0400 | [-0.3186, -0.1622] |
| Peer integration | Indirect at x1: F(x1,x1) − F(x1,x0) | -0.2155 | 0.0375 | [-0.2944, -0.1480] |
| Peer integration | Direct at distribution x0: F(x1,x0) − F(x0,x0) | -0.3238 | 0.1396 | [-0.6011, -0.0462] |
| Peer integration | Direct at distribution x1: F(x1,x1) − F(x0,x1) | -0.3029 | 0.1308 | [-0.5631, -0.0439] |
| Peer integration | Total: F(x1,x1) − F(x0,x0) | -0.5393 | 0.1373 | [-0.8120, -0.2720] |
| Faculty relationship quality | Indirect at x0: F(x0,x1) − F(x0,x0) | -0.1295 | 0.0416 | [-0.2172, -0.0548] |
| Faculty relationship quality | Indirect at x1: F(x1,x1) − F(x1,x0) | -0.1325 | 0.0432 | [-0.2252, -0.0552] |
| Faculty relationship quality | Direct at distribution x0: F(x1,x0) − F(x0,x0) | 0.0781 | 0.1984 | [-0.3032, 0.4650] |
| Faculty relationship quality | Direct at distribution x1: F(x1,x1) − F(x0,x1) | 0.0751 | 0.1907 | [-0.2937, 0.4459] |
| Faculty relationship quality | Total: F(x1,x1) − F(x0,x0) | -0.0543 | 0.1962 | [-0.4299, 0.3241] |

Bootstrap procedure: 5,000 person-level resamples per relationship model, NumPy default_rng seed 20260913. Multinomial frequency weights represent sampling 3,014 persons with replacement. In each resample both component models, including NB dispersion, were refitted, and predictions were averaged using the resample's covariate distribution. The generator was reset to the same seed for the two relationship measures, producing the same sequence of person resamples. All 5,000 fits per relationship measure completed without recorded failures. Confidence limits are empirical 2.5th and 97.5th percentiles; standard errors are the sample standard deviations of the bootstrap estimates. The earlier linear bootstrap and this nonlinear bootstrap need not use identical draws.

Implementation and checks: nonlinear_sensitivity.py implements multinomial maximum likelihood with Newton steps and step halving and NB2 estimation with iterative coefficient fitting and profile optimization of dispersion. The archived numerical checks include near-zero NB score gradients, probabilities summing to one, the contrast decomposition identities, and zero indirect contrasts when the relevant exposure or mediator contributions are set to zero. Component coefficients were checked against SPSS output, and combined point estimates were separately reproduced in SPSS. Full nonlinear bootstrap intervals were calculated in Python, not in SPSS. A subsequent execution of the notebook reproduced the archived point estimates without rerunning the bootstrap. These are implementation and reproducibility checks, not an independent external statistical review.

The estimates are standardized associational contrasts. Causal identification assumptions, including absence of unmeasured confounding, are not established. Agreement in direction with the linear analyses supports sensitivity assessment but does not show that either specification is correct or that the estimands are identical.

Reproduction materials: nonlinear_sensitivity.py, nonlinear_sensitivity_results.json, nonlinear_sensitivity_QA.json and Python_Auswertungen_Revision.ipynb. Data source: NEPS_SC5_Analysedaten.xlsx, sheet Paper1_N3014. The archived SHA-256 is 7c7859f10e7ff846349b841687f484a7136abe0627453fac172ab43f09ffcac9. Individual-level data remain subject to NEPS access conditions.

# Supplementary Figure S1

*Supplementary Figure S1. Assumed structure for each separately fitted mediation model. X represents either peer integration or faculty relationship quality at W4 (2012), M loneliness at W6 (2013/2014), and Y mentally unhealthy days at W10 (2016). C denotes adjusted covariates; U denotes potential unmeasured common causes, including prior mental health. Grey dashed arrows indicate possible unmeasured confounding, not nonsignificant estimates. The diagram does not establish that covariate adjustment identifies causal effects and does not exhaust all possible confounding or selection mechanisms.*
